# Utilization of Response Surface Methodology in Optimization of Polysaccharides Extraction from Vietnamese Red *Ganoderma lucidum* by Ultrasound-Assisted Enzymatic Method and Examination of Bioactivities of the Extract

**DOI:** 10.1155/2021/7594092

**Published:** 2021-02-11

**Authors:** Dat Tran Do, Dang Hoang Lam, Tai Nguyen, Tran Thi Phuong Mai, Le Thao My Phan, Hoai Thanh Vuong, Duc Viet Nguyen, Ngo Thi Thuy Linh, Minh Nam Hoang, Thanh Phong Mai, Huu Hieu Nguyen

**Affiliations:** ^1^VNU-HCMC Key Laboratory of Chemical Engineering and Petroleum Processing (CEPP Lab), Ho Chi Minh City, Vietnam; ^2^Faculty of Chemical Engineering, Ho Chi Minh City University of Technology, 268 Ly Thuong Kiet Street, Ward 14, District 10, Ho Chi Minh City, Vietnam; ^3^Vietnam National University Ho Chi Minh City, Linh Trung Ward, Thu Duc District, Ho Chi Minh City, Vietnam

## Abstract

Red *Ganoderma lucidum (G. lucidum)* is a popular medicinal herb commonly used in Vietnamese traditional remedies due to its potential value for health. In this study, polysaccharides were extracted from *G. lucidum* using ultrasound-assisted enzymatic extraction method. The response surface methodology and Box–Behnken design were employed to investigate the effects of pH, extraction temperature, extraction time, and ultrasonic power on the content of polysaccharides. Based on ultraviolet-visible spectroscopy analysis, the highest content of polysaccharides in the extract was 32.08 mg/g under optimum experimental parameters including enzyme concentration of 3%, pH of 5.5, extraction temperature of 45°C, extraction time of 30 min, and ultrasonic power of 480 W. The Fourier-transform infrared spectroscopy was also used to identify the functional groups in the extracts. The molecular weights of polysaccharides were determined by gel permeation chromatography. The obtained extract was then evaluated for anticancer activities by using (3-(4,5-dimethylthiazol-2-yl)-2,5-diphenyltetrazolium bromide) assay, showing the anticancer activities with the half-maximal inhibitory concentration value of more than 512 *μ*g/mL. This result suggested that UAEE could be considered as an appropriate and effective extraction method for bioactive crude polysaccharides from *G. lucidum.*

## 1. Introduction

*Ganoderma lucidum* (*G*. *lucidum*), which is known as *Lingzhi* in China, is a basidiomycete fungus belonging to the Polyporaceae family. It is widely used in China as a remedy for minor health disorders to promote vitality and longevity. In East Asia, it has been long used to treat many diseases such as nephritis, chronic hepatitis, gastric ulcers, arthritis, neurasthenia, bronchitis, asthma, and hypertension in oriental countries [1–5]. Recent studies on *G*. *lucidum* have shown that it has numerous bioactivities, including anticancer, antioxidant, and immunomodulating effects [6, 7]. To gain insights into the mechanisms of polysaccharides and triterpenoids for health benefits, their effects on various human cell lines have been investigated extensively [8–13]. With the objective of obtaining extracts with higher yields and lower costs, various methods of extraction have been developed. Among them, the ultrasound-assisted extraction (UAE) method and enzyme-assisted extraction (EAE) method have become ideal alternatives to traditional techniques because of their efficiency and environment friendliness. It is reported that while EAE requires long extraction time, the capability of enzyme could be enhanced by ultrasonic wave. Therefore, ultrasonic-assisted enzymatic extraction (UAEE) could be used for polysaccharide extraction from *G*. *lucidum* so that a higher content of polysaccharide can be achieved in shorter extraction time.

Composed of monosaccharide unit long chains linked by glycosidic bonds, polysaccharides are polymeric carbohydrate molecules [14]. Gel permeation chromatography (GPC) was widely applied for characterization of polysaccharides with identification of molecular weight averages and molecular weight distributions offering numerous advantages [15, 16]. In this study, polysaccharides were extracted from *G*. *lucidum* using UAEE method. Response surface methodology (RSM) was used to optimize the extraction conditions including pH, extraction temperature, extraction time, and ultrasonic power. Under optimum extraction conditions, the extract obtained was then investigated by using the Fourier-transform infrared spectroscopy (FTIR) and the GPC, followed by the anticancer activity evaluation for three lines of KB, HepG2, and Lu cancer cells.

## 2. Materials and Methods

### 2.1. Material and Chemicals

Dried fruiting bodies of Vietnamese Red *G. lucidum* in maturity stage with mature spores were stored in closed plastic bag and were supplied by Linhchivina Co., JSC (Vietnam).

Viscozyme was purchased from Novozymes, Denmark, chitinase was purchased from Sigma-Aldrich, and 95.0% and 99.5% ethanol were purchased from Chemsol. 99.0% D-glucose, 99.5% sodium hydrogen phosphate (Na_2_HPO_4_), 99.5% sodium tetraborate decahydrate (Na_2_B_4_O_7_.10 H_2_O), 37.0% hydrochloric acid (HCl), 99.5% phenol, 98.0% sulfuric acid, 99.0% citric acid, and 99.7% ascorbic acid were purchased from Xylong, China.

### 2.2. Extraction Methods

**Figure 1:**
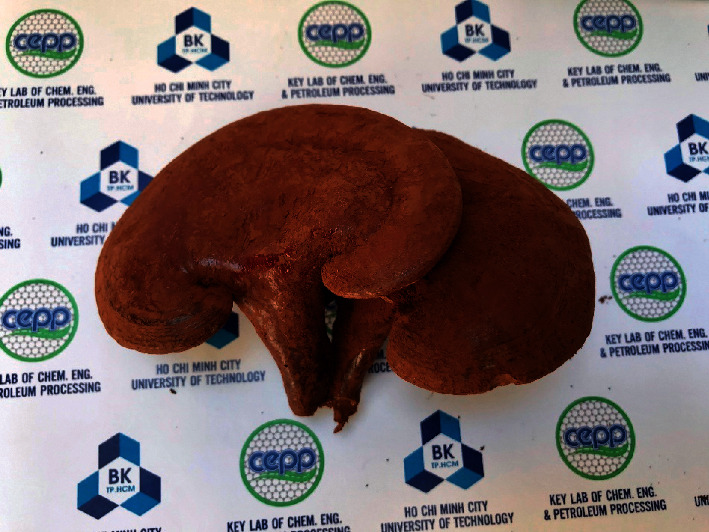
Image of Vietnamese red *G. lucidum*.

5.0 g of fungus powder was dispersed in 100 mL of distilled water, followed by the addition of 100 *μ*L of the mixture of enzymes including vicozyme and chitinase with a ratio of 1 : 1. The extraction conditions including pH value, extraction temperature, extraction time, and ultrasonic power were set based on the experimental design. After filtration, the solvent was partially removed by using vacuum evaporation at 65–70°C. The concentration was then precipitated with the addition of 100 mL 99.5% ethanol at 4°C in 12 hours. Next, the mixture was centrifuged, and the precipitate was collected before being dried to obtain the crude polysaccharides. According to reported research, the crude polysaccharides were preliminarily purified using the Sevag method for deproteinization of the extract [17]. First, the Sevag solution including *n*-butanol and chloroform with the ratio of 1 : 4 was prepared. After the addition of the Sevag solution to obtain polysaccharide aqueous extract, the mixture was gently shaken and allowed to be settled for the phases to separate. The supernatant was collected and precipitated by adding 96% ethanol with a proportion of 1 : 4 at 4°C for 24 hours and then centrifuged at 12000 rpm for 20 min. Subsequently, the achieved precipitate was dried at 60°C. The procedure was repeated 3 times or more for the removal of the protein.  Figure 1 shows the image of red *G. lucidum* provided by Linhchivina Co., JSC (Vietnam).

### 2.3. Box–Behnken Design and Statistical Analysis

From the result of single factor experiments on the content of polysaccharides, four factors having the most influence on the content of polysaccharides and the experimental variable ranges of these factors were determined according to previous studies [18]. The experiments were conducted based on three-level Box–Behnken design. Four independent variables including pH, extraction temperature, extraction time, and ultrasonic power were designated as *X*_1_, *X*_2_, *X*_3_, and *X*_4_, respectively.  Table 1 shows the independent variables and their levels.

### 2.4. Determination of the Polysaccharide Content

The phenol-sulfuric acid colorimetric method was used to determine content of polysaccharides with D-glucose as standard solution. Six standard D-glucose solutions including 50, 100, 200, 300, 400, and 500 *μ*g/mL were prepared from D-glucose solution with concentration of 1000 *μ*g/mL. Then, 1 mL of each standard solution was removed and transferred to a volumetric flask (20 mL), followed by the addition of 1 mL of 5% phenol solution and 5 mL of 98% concentrated sulfuric acid. In addition, 1 mL of distilled water was added to 1 mL of 5% phenol solution and 5 mL of 98% concentrated sulfuric acid to prepare blank solution whereas the extract solution was prepared by the addition of 1 mL of the extract, 1 mL of 5% phenol solution, and 5 mL of 98% concentrated sulfuric acid. After 30 min, the measurement of absorbance was conducted at 488 nm. The content of polysaccharides was determined according to the absorbance of the extract solution and baseline.

### 2.5. Fourier-Transform Infrared Spectroscopy Analysis

The FTIR measurements were carried out at VNU-HCMC Key Laboratory of Chemical Engineering and Petroleum Processing (CEPP Lab), Ho Chi Minh City University of Technology-Vietnam National University Ho Chi Minh City (HCMUT-VNUHCM), with the FTIR spectrometer Alpha II from Bruker, Germany. For FT-IR measurement, the polysaccharides were ground with KBr powder and pressed into pellets. The frequency range used was 4000–400 cm^−1^ to detect functional groups.

### 2.6. Gel Permeation Chromatography

The molecular weights of polysaccharides were determined by the GPC, in combination with a high-performance liquid chromatography instrument (Angilent1100, USA) equipped with an Ultrahydrogel column. The experiments were conducted at the Central Laboratory for Analysis (CLA), Ho Chi Minh City University of Science-Vietnam National University Ho Chi Minh City (HCMUS-VNUHCM).

The column temperature was maintained at 30°C. Samples were filtered through a 0.45 *μ*m filter and the injection volume was 20 *μ*L. The mobile phase was 0.1 M in potassium nitrate, and the flow rate was 1.0 mL/min and detected by a refractive index detector [19, 20].

### 2.7. Anticancer Activities of the Extract

The anticancer capacity of the extract was evaluated by using 3-(4,5-dimethylthiazol-2-yl)-2,5-diphenyltetrazolium bromide (MTT) assay against cell lines including KB oral squamous cell carcinoma, liver cancer cell line HepG2, and lung cancer cell line Lu-1. The experiments were conducted at Laboratory of Applied Biochemistry-Institute of Chemistry-Vietnam Academy of Science and Technology. 190 *μ*L of the cell solution was mixed with 10 *μ*L of the extract sample. The control tests included the positive control which contained the cancer cells and the negative control which was the cultural medium after 72 hours of incubation.

Each sample was then added to 10 *μ*L of 5 mg/mL MTT solution and incubated for 4 hours. Finally, the sample was separated from media and formazan crystal was dissolved into 100 *μ*L of absolute DMSO solution. The absorbance was measured at 540 nm by Genios Tecan spectrograph and the IC_50_ value was calculated by the % cell inhibition.

## 3. Results and Discussion

### 3.1. Experimental Design

Twenty-seven randomized experimental runs were conducted.  Table 2 presents the variable conditions used in each experimental assay.

By multiple regression analysis on the experimental data, the predicted response on content of polysaccharides and the test variables were related by equation (1) showing quadratic polynomial model based on actual value:

(1) $$\begin{array}{c} Y=32.4+0.67X_{1}+1.58X_{2}+0.77X_{3}-0.85X_{4}-0.51X_{1}X_{2}-0.07X_{1}X_{3}-0.30X_{1}X_{4}-0.21X_{2}X_{3}-0.67X_{2}X_{4}-0.34X_{3}X_{4}-5.43X_{1}^{2}-5.86X_{2}^{2}-4.11X_{3}^{2}-3.47X_{4}^{2}, \end{array}$$

where *Y* (mg/g) is the content of polysaccharides and four variables including *X*_1_, *X*_2_, *X*_3_, and *X*_4_ are pH, extraction temperature (°C), extraction time (min), and ultrasonic power (W).

Table 3 illustrates the analysis of variance (ANOVA) for the response surface quadratic model, and *F* value and *p* value were used to check the statistical significance of the regression equation. Evident from Fisher's *F* test with a high model *F* value (30.95) but a low *p* value (*p* < 0.0001), ANOVA of quadratic regression model showed that the model was highly significant and had a good fit of the model. The determination coefficient (*R*^2^ = 0.97) was used to evaluate the goodness-of-fit of the model, showing that only 3.14% of the total variations could not be explained by the model. Moreover, Pre-*R*^2^ is 0.8468, which was smaller and very closed to Adj-*R*^2^, which was of 0.94 (Adj-*R*^2^-Pre-*R*^2^ <0.2), presenting a high correlation degree between the observed and predicted data from the regression model [21]. In addition, the low value of coefficient of the variation (C.V. %) of 3.59% for the content of polysaccharides represented the dispersion of data points was around the mean and had a good reliability. The linear coefficients *X*_1_, *X*_2_, *X*_3_, *X*_4_ and the quadratic coefficients *X*_1_^2^, *X*_2_^2^, *X*_3_^2^, and *X*_4_^2^ had significant effects on content of polysaccharides (*p* < 0.05). The other coefficient influences on content of polysaccharides were not significant (*p* > 0.05).

**Figure 2:**
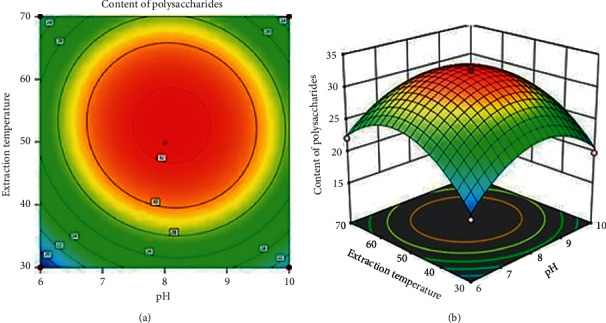
Simultaneous effect of pH and extraction temperature on the content of polysaccharides.

**Figure 3:**
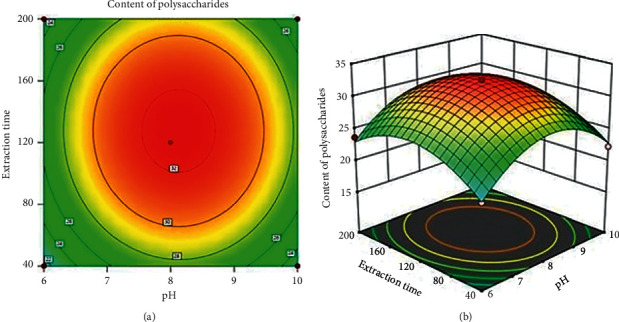
Simultaneous effect of pH and extraction time on the content of polysaccharides.

**Figure 4:**
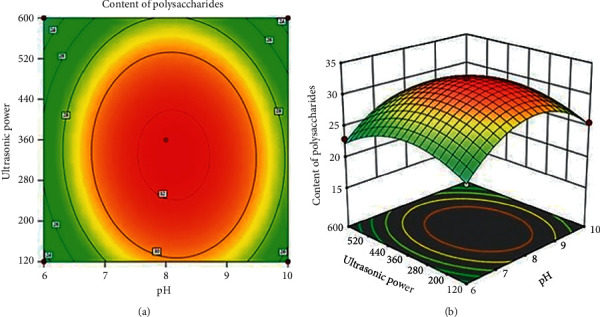
Simultaneous effect of pH and ultrasonic power on the content of polysaccharides.

**Figure 5:**
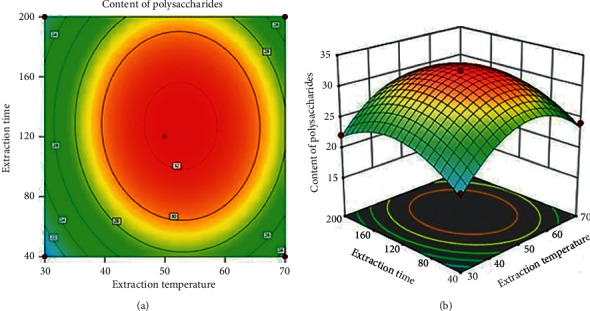
Simultaneous effect of extraction temperature and extraction time on the content of polysaccharides.

**Figure 6:**
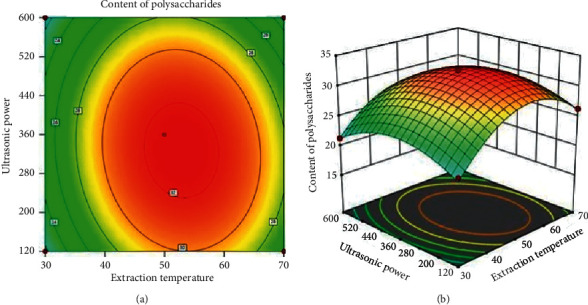
Simultaneous effect of extraction temperature and ultrasonic power on the content of polysaccharides.

**Figure 7:**
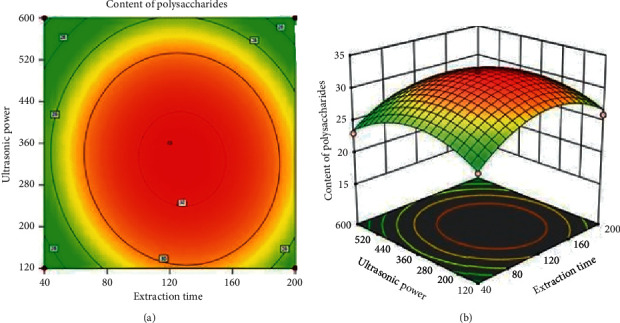
Simultaneous effect of extraction time and ultrasonic power on the content of polysaccharides.

The impacts of independent variables and their mutual interaction on the yield of polysaccharides can be evaluated and visualized by response surface and contour plots as shown in Figures 2–7 by employing the software Design-Expert (Version 11.0, Stat-Ease Inc., Minneapolis, MN, USA). It could be deduced based on Figure 1 that the optimum conditions for extraction procedure were predicted including extraction time of 127.69 min, extraction temperature of 52.78^o^C, ultrasonic power of 325.99 W, and pH value of 8.12.

### 3.2. Simultaneous Effect of Extraction Conditions

#### 3.2.1. Effect of pH and Extraction Temperature on the Content of Polysaccharides

Figure 2 shows the simultaneous effects of pH and extraction temperature on content of polysaccharides when ultrasonic power was kept with the value of 360 W and extraction time was of the value of 120 min. It is clearly seen that, with the increase of extraction temperature and pH value, content of polysaccharides was increased dramatically. This could be explained that when the pH and the extraction temperature were not too high, the impact of enzyme and high ultrasonic power efficiently broke the structure of the cell wall, leading to the diffusion of content of polysaccharides into solvent. However, at high pH (>8.2) and high temperature (>52^o^C), the yield of polysaccharides experienced a decrease because polysaccharides were reported to be more likely to be hydrolyzed under these conditions [22, 23]. This result is suitable with a previous study [24].

#### 3.2.2. Effect of pH and Extraction Time on the Content of Polysaccharides

Figure 3 illustrates the simultaneous effects of pH and extraction time on content of polysaccharides while the ultrasonic power was of 360 W and extraction temperature was of 50°C. It is clearly seen that the increase of pH value and extraction time led to the increase of the content of polysaccharides. The content of polysaccharides was slightly decreased when the pH value reached 7.8 with the extraction time of over 120 min. It is stated that although longer extraction time could facilitate the extraction process, this could lead to processes of oxidation and degradation of polysaccharide [25, 26]. This result, therefore, can be found similar to the other studies [23, 24, 27].

#### 3.2.3. Effect of pH and Ultrasonic Power on the Content of Polysaccharides

Figure 4 demonstrates the simultaneous effects of pH and ultrasonic power on content of polysaccharides while the extraction time was of 120 min and extraction temperature was of 50^o^C. The high content of polysaccharides obtained at ultrasonic power ranged from 280 to 400 W and pH ranged between 7.5 and 8.5. The increase in ultrasonic power and pH level could facilitate the destruction of cell wall, leading to the diffusion of solvent through cell walls to release polysaccharides. However, when the ultrasonic power was higher than 400 W, enzyme could be inactivated and the cavitation effect could be weakened [28, 29]. This result is in agreement with the result reported in previous research [23, 24, 26].

#### 3.2.4. Effect of Extraction Temperature and Extraction Time on the Content of Polysaccharides

Figure 5 presents the mutual interactions of extraction temperature and extraction time on content of polysaccharides., where the ultrasonic power was kept at 360 W and pH value was of 8.0. As shown in Figure 5, the content of polysaccharides significantly increased as the extraction time was raised from 40 to 120 min and the extraction temperature increased from 30 to 48^o^C. The longer the extraction time was, the higher the diffusion capacity of the active ingredient was. Nevertheless, when extraction temperature was over 48^o^C and extraction time longer than 120 min, the content of polysaccharides decreased gradually. This could be explained by the fact that when extraction time and extraction temperature exceed the values, the hydrolysis of polysaccharides tends to happen vigorously, leading to a decrease in the content of polysaccharides according to reported experiments [20, 23, 27].

#### 3.2.5. Effect of Extraction Temperature and Ultrasonic Power on the Content of Polysaccharides

Figure 6 describes the simultaneous effects of extraction temperature and ultrasonic power on content of polysaccharides. The content of polysaccharides increased considerably in the extraction temperature which ranged from 45 to 55°C and ultrasonic power which ranged between 220 W and 400 W. Earlier reports indicated that wave could improve mass transfer, enhancing the efficiency of substrate delivery to the active sites of the enzymes [23, 27, 30–32].

#### 3.2.6. Effect of Extraction Time and Ultrasonic Power on the Content of Polysaccharides

The simultaneous effects of extraction time and ultrasonic power on content of polysaccharides are as shown in Figure 7, where the extraction temperature was kept at 50^o^C and pH value was of 8.0. From Figure 7, it is clear that the content of polysaccharides was low when extraction time ranged between 40 and 80 min and ultrasonic power ranged between 120 and 240 W. The increase in extraction power facilitates the disruption of the cell walls, enhances the presented compounds solubility, and increases the extraction yield [20]. Moreover, higher ultrasonic power and longer extraction time lead to slight increase in the content of polysaccharides. It was reported that prolonged extraction time could cause saturation between solvent and substrates while polysaccharides could be degraded under the influence of high ultrasonic power [26, 27, 33].

To meet the actual conditions operability, predicted and experimental optimum extraction conditions are as shown in Table 4. Under these conditions, the experimental content of polysaccharides was 32.08 mg/g, which was in good agreement with the prediction of 32.63 mg/g.

### 3.3. Fourier-Transform Infrared Spectroscopy Analysis

**Figure 8:**
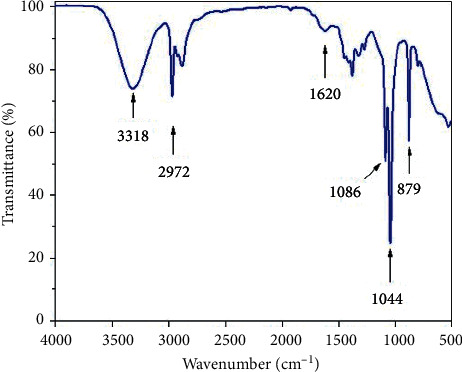
FT-IR spectra of polysaccharides.

Figure 8 illustrates the FT-IR spectrum of polysaccharides in order to confirm characteristics of polysaccharides. The broad band around 3318 cm^−1^ was caused by O-H stretching vibration. Meanwhile, the band at 2972 cm^−1^ corresponded to the C-H absorption of –CH_2_ in polysaccharides structures. The peak at 1044 cm^−1^ revealed the presence C=O stretching vibration. The existence of a weak absorption band at 1620 cm^−1^ was typical of glycosidic bonds in polysaccharides structure [34]. In addition, glycosidic bonds might be also represented in the relatively weak absorption peaks at 1620 cm^−1^ [35]. Moreover, the stretching vibration of 879 cm^−1^ referred to the absorption of *β*-D-glucose with the pyranose form [35, 36]. The other bands observed at 1086, 1044, and 879 cm^−1^ were characteristic absorptions of to *β*-(1,3), (1,6)-D-glucan [37].

### 3.4. Gel Permeation Chromatography

The molecular weights (Mw), number average molecular weights (Mn), and corresponding polydispersity index (PDI) were determined by GPC. The results showed that the Mw and Mn values were of 2.6843 × 10^3^ and 1.6961 × 10^3^ Da, respectively, with the PDI value of 1.5826. The PDI is a measure of broadness of molecular weight distribution and the PDI of a polymer is calculated as the ratio of weight average by number average molecular weight [38]. It was stated that the lower Mw polysaccharides would have fairly narrow molecular weight distribution (MWD) and PDI of 1 to 1.5, whereas the higher Mw polysaccharides are generally regarded as having broad MWD and large PDI [39]. The PDI value of 1.5826, which was higher than 1.5, may be caused by natural product as most natural occurring polysaccharides illustrated high PDI value [14, 40]. Basically, MWD refers to the amounts of component polymers making up a polymer and is essential for microstructural quality description of a polymer [41, 42]. In addition, according to previous studies, polysaccharide was recorded with the Mn value of 1013 × 10^3^ Da extracted from the fruiting bodies of *Ganoderma atrum* while from *G. lucidum* by using ultrasonic-aid extraction, polysaccharides with the Mn values of 1.926 × 10^3^ Da and 1086 × 10^3^ Da could be extracted [43, 44]. These reported results were higher than that obtained in this study. Therefore, it could be suggested that the employment of ultrasound wave and enzyme could have influence on structures of long chain polysaccharides in the sample.

### 3.5. Anticancer Activities of Extracts

The test results of anticancer activity of the extract from *G. lucidum* on three lines of KB, HepG2, and Lu cancer cells are illustrated in Table 5. It can be seen from Table 5 that content of polysaccharides did not have significant influence on these cancer cell lines. At the same time, ellipticine showed strong cytotoxic activities with low IC_50_ values (<0.50 *μ*g/mL). Nevertheless, according to previous study, the anticancer abilities of polysaccharides extracted from *G. lucidum* were not based on direct cytotoxicity effect of polysaccharides on cell lines. It is reported that polysaccharides showed the low anticancer activities against breast cancer cell lines, with a concentration of 50 *μ*g/mL, but only 4.81% was inhibited. However, in the presence of polysaccharides and macrophages (cells of the immune system), polysaccharides were able to enhance the activity of macrophages and the inhibitory ability of cancer cells was significantly increased to 38% at the same concentration of 50 *μ*g/mL [44]. Therefore, polysaccharides could be regarded as potential anticancer agents due to their boosting of the immune system ability. Meanwhile, cytotoxic effects on cancer cell lines remained unclear. Therefore, polysaccharides have poor cytotoxic effects on three cancer cell lines including KB, HepG2, and Lu [44].

## 4. Conclusions

In this study, by using UAEE, polysaccharides were extracted from *G. lucidum* with the employment of the RSM and Box–Behnken design under the researched effects of pH, extraction temperature, extraction time, and ultrasonic power. According to ultraviolet-visible spectroscopy analysis, the maximum content of polysaccharides in the extract was 32.08 mg/g under optimal conditions of enzyme concentration of 3%, pH of 5.5, extraction temperature of 45°C, extraction time of 30 min, and ultrasonic power of 480 W. By using GPC technique, the Mw and Mn values of polysaccharides in the extract were of 2.6843 × 10^3^ and 1.6961 × 10^3^ Da, respectively, with the PDI value of 1.582. The extract under these conditions was then evaluated for anticancer activities by (3-(4,5-dimethylthiazol-2-yl)-2,5-diphenyltetrazolium bromide) assay, showing that the IC_50_ values were of more than 512 *μ*g/mL for three cancer cell lines. Therefore, UAEE could be considered as an appropriate and effective extraction method for polysaccharides from *G. lucidum*.

## Figures and Tables

**Table 1:** Independent variables and their levels.

Independent variables	Unit	Code	Levels		
			−1	0	1
pH		*X*_1_	6	8	10
Extraction temperature	°C	*X*_2_	30	50	70
Extraction time	min	*X*_3_	40	120	200
Ultrasonic power	W	*X*_4_	120	360	600

**Table 2:** Factors and levels for RSM, and Box–Behnken design with the independent variables.

Run *X*	*X*_1_	*X*_2_	*X*_3_	*X*_4_	*Y*	
					Experimental	Predicted
1	0	0	−1	−1	24.3	24.5
2	−1	−1	0	0	17.5	18.3
3	0	0	0	0	32.5	32.4
4	−1	0	0	−1	23.2	23.3
5	0	0	1	−1	26.0	26.7
6	1	1	0	0	22.4	22.9
7	0	1	−1	0	24.2	23.4
8	−1	1	0	0	22.2	22.5
9	−1	0	0	1	23.0	22.3
10	1	0	0	−1	25.7	25.3
11	0	1	0	−1	26.3	26.1
12	−1	0	−1	0	21.2	21.3
13	1	−1	0	0	19.8	20.7
14	0	0	0	0	32.0	32.4
15	0	−1	1	0	22.2	21.8
16	−1	0	1	0	23.7	23.1
17	1	0	1	0	24.6	24.3
18	0	−1	0	−1	22.4	21.5
19	0	1	1	0	25.0	24.6
20	1	0	−1	0	22.3	22.9
21	0	−1	−1	0	20.5	19.8
22	0	1	0	1	22.6	23.1
23	1	0	0	1	24.2	23.1
24	0	0	0	0	32.6	32.4
25	0	0	1	1	23.4	24.5
26	0	0	−1	1	23.1	23.5
27	0	−1	0	1	21.3	21.3

**Table 3:** ANOVA for the second-order polynomial model.

Source	Sum of squares	Degree of freedom	Mean squares	*F* value	*p* value	
Model	322.30	14	23.02	30.95	<0.0001	Significant
*X*_1_-pH	5.52	1	5.52	7.52	0.0185	
*X*_2_-extraction temperature	29.94	1	29.94	40.24	<0.0001	
*X*_3_-extraction time	7.16	1	7.16	9.63	0.0091	
*X*_4_-ultrasonic power	8.58	1	8.58	11.53	0.0053	
*X*_1_ *X*_2_	1.04	1	1.04	1.40	0.2603	
*X*_1_ *X*_3_	0.0172	1	0.0172	0.0232	0.8816	
*X*_1_ *X*_4_	0.3634	1	0.3634	0.4885	0.4979	
*X*_2_ *X*_3_	0.1767	1	0.1767	0.2376	0.6347	
*X*_2_ *X*_4_	1.79	1	1.79	2.40	0.1470	
*X*_3_ *X*_4_	0.4639	1	0.4639	0.6236	0.4450	
*X*12	156.96	1	156.96	211.00	<0.0001	
*X*22	183.41	1	183.41	246.55	<0.0001	
*X*32	89.93	1	83.93	120.89	<0.0001	
*X*42	64.35	1	64.35	86.51	<0.0001	
Residual	8.93	12	0.7439			
Lack of fit	8.73	10	0.8730	8.88	0.1053	Not significant
Pure error	0.1965	2	0.0863			
Cor total	331.23	26				
*R*2	0.9730	SD	0,0863			
Adj-*R*^2^	0.9416	C.V %	3.59			
Pred-*R*^2^	0.8468					
Adep. precision	21.8652					

**Table 4:** Predicted and experimental optimum extraction conditions.

	pH	Extraction temperature (°C)	Extraction time (min)	Ultrasonic power (W)	Content of polysaccharides (mg/g)
Predicted conditions	8.12	52.78	127.69	325.99	32.63
Experimental conditions	8.00	53	128.00	360.00	32.08

**Table 5:** IC_50_ values of the extract.

	IC_50_ value (*μ*g/mL)		
	KB	HepG2	Lu
The extract	>512	>512	>512
Ellipticine	0.45	0.38	0.41

## Data Availability

The data used to support the findings of this study are included within the article.
